# Heat-killed endophytic bacterium induces robust plant defense responses against important pathogens

**DOI:** 10.1038/s41598-021-91837-5

**Published:** 2021-06-09

**Authors:** Roxana Portieles, Hongli Xu, Qiulin Yue, Lin Zhao, Dening Zhang, Lihua Du, Xiangyou Gao, Jingyao Gao, Nayanci Portal Gonzalez, Ramon Santos Bermudez, Orlando Borrás-Hidalgo

**Affiliations:** 1 Joint R&D Center of Biotechnology, RETDA, Yota Bio-Engineering Co., Ltd., 99 Shenzhen Road, Rizhao, 276826 Shandong People’s Republic of China; 2 State Key Laboratory of Biobased Material and Green Papermaking, Shandong Provincial Key Lab of Microbial Engineering, Qilu University of Technology (Shandong Academic of Science), Jinan, People’s Republic of China; 3 School of Biological Science and Technology, University of Jinan, No. 336, West Road of Nan Xinzhuang, Jinan, 250022 Shandong People’s Republic of China

**Keywords:** Microbiology, Plant sciences

## Abstract

Stress caused by pathogens strongly damages plants. Developing products to control plant disease is an important challenge in sustainable agriculture. In this study, a heat-killed endophytic bacterium (HKEB), *Bacillus aryabhattai*, is used to induce plant defense against fungal and bacterial pathogens, and the main defense pathways used by the HKEB to activate plant defense are revealed. The HKEB induced high protection against different pathogens through the salicylic and jasmonic acid pathways. We report the presence of gentisic acid in the HKEB for the first time*.* These results show that HKEBs may be a useful tool for the management of plant diseases.

## Introduction

In nature, plants are constantly affected by different biotic and abiotic stresses^1,2^. The effects of diseases caused by plant pathogens are among the main limiting factors of crop yields, resulting in losses of 10–30% every year in different important crops^3^. The control of plant diseases should involve integral sustainable management where farmers combine different genetic, biological, chemical and agricultural practices^4^. Sustainable management systems allow plants to be protected from disease through environmentally friendly approaches while achieving adequate yields^5^.

Plant species have evolved an innate immune system with two levels of pathogen recognition^6^. The levels differ in terms of localization, pattern recognition and defense response development. The first level of recognition is established in the plasma membrane or apoplastic space through pattern recognition receptors, such as receptor-like kinases or receptor-like proteins. These receptors recognize microbe-associated molecular patterns (MAMPs), damage-associated molecular patterns (DAMPs)^7^ and pathogen-associated molecular patterns (PAMPs)^8^. This recognition enables a series of molecular events, such as oxidative burst, nitric oxide production, callose deposition, and the induction of transcription factors related to plant defense reactions via a complex stream of mitogen-activated protein kinases^9^, to occur. The second level of recognition occurs in the cytoplasm through nucleotide-binding site leucine-rich repeat (NB-LRR) receptors that detect pathogen effector proteins^6,10,11^.

There are several different types of MAMPs in bacteria, including flagellin, which is the main protein of flagella^12,13^, and the elongation factor Tu^14,15^. Other MAMPs include lipoproteins, lipopeptides, porins, peptidoglycan, lipoteichoic acid, lipoarabinomannan, mycolic acids, mannose-rich glycans, N-formylmethionine, glycolipids and lipopolysaccharide, the last of which is present in the outer membrane of the bacterial cell wall^16–20^.

Previous studies have shown that peptidoglycan triggers the defense response in *Arabidopsis thaliana*^19^, tobacco^21^, tomato^22^ and rice^23^. In addition, lipopolysaccharides activate defense responses in plants^24^. Treatment with lipopolysaccharides in *A. thaliana* induces nitric oxide (NO) synthase, which plays an important role in defense gene expression and resistance to pathogenic bacteria^18,25,26^.

Heat-killed bacteria have been proposed as a good option for the treatment of different diseases. Heat-killed lactic acid bacteria possess immunomodulatory functions, providing the advantages of a longer product shelf life, easier storage, and more convenient transportation. These cells have immunomodulatory ability via increased cytokines triggering the immune response^27,28^. However, studies on the role of heat-killed bacteria in plant defense activation are lacking.

Previous studies have shown that some endophytic bacterial species activate a systemic defense against different types of plant pathogens^29^. A number of plant endophytic bacteria protect plants from soil-borne pathogens, inducing systemic resistance in aerial plant parts. For example, *A. thaliana* plants treated with *Bacillus pumilus* strain SE34 showed reduced disease severity and symptom development in relation to *cucumber mosaic virus*^30^. The induction of systemic resistance was mediated by plant signaling molecules such as jasmonic acid (JA) and ethylene^30^. However, the biocontrol effect of this bacterium against *A. thaliana* root infection by *Pseudomonas syringae* was attributed to its abilities to form biofilms and to produce surfactin^31^. Additionally, in *A. thaliana* (Col-0) plants exposed to a *Bacillus subtilis-*derived elicitor, acetoin triggered a strong defense response to *P. syringae* pv. tomato DC3000 through salicylic acid/ethylene, whereas JA was not essential^32^. Although endophytic microbes can establish an interesting and beneficial alliance during plant interactions, little is known about how MAMPs from heat-killed endophytic bacteria (HKEBs) could be used to activate plant defense. The use of HKEBs or their fractions or purified components with innate immune regulatory functions in different areas creates the possibility of using this approach to activate plant defense against important diseases^33–37^.

Interesting results have been obtained using bioactive compounds and natural products under field conditions^38–41^. These compounds can induce important reactions, trigger endogenous plant defense responses, inhibit pathogen colonization and proliferation and facilitate sustainable and healthy agriculture^42–44^. The use of environmentally friendly products is an appropriate practice for avoiding the negative impact of chemical pesticides^45^. The use of HKEBs is an interesting option for activating plant defense against different diseases.

*Botrytis cinerea,* one of the most notorious cosmopolitan fungi and the second most important phytopathogenic fungus, is a model for studying the infection process of necrotrophic fungi^46^, while *P. syringae* pv. tomato, a phytopathogenic bacterial species that includes pathogenic strains of a wide variety of plant species^47^, has been used as a model to elucidate several key interactions between plants and biotrophic pathogens^6^. Hence, our main aim was to evaluate HKEB as inducers of plant defense against such necrotrophic and biotrophic pathogens. We used different functional evaluations to detect and show the high induction of plant defense by HKEB. In addition, we identified the key molecules in HKEB that are involved in this activation.

## Results

### Identification of the endophytic bacterial strain

**Figure 1:**
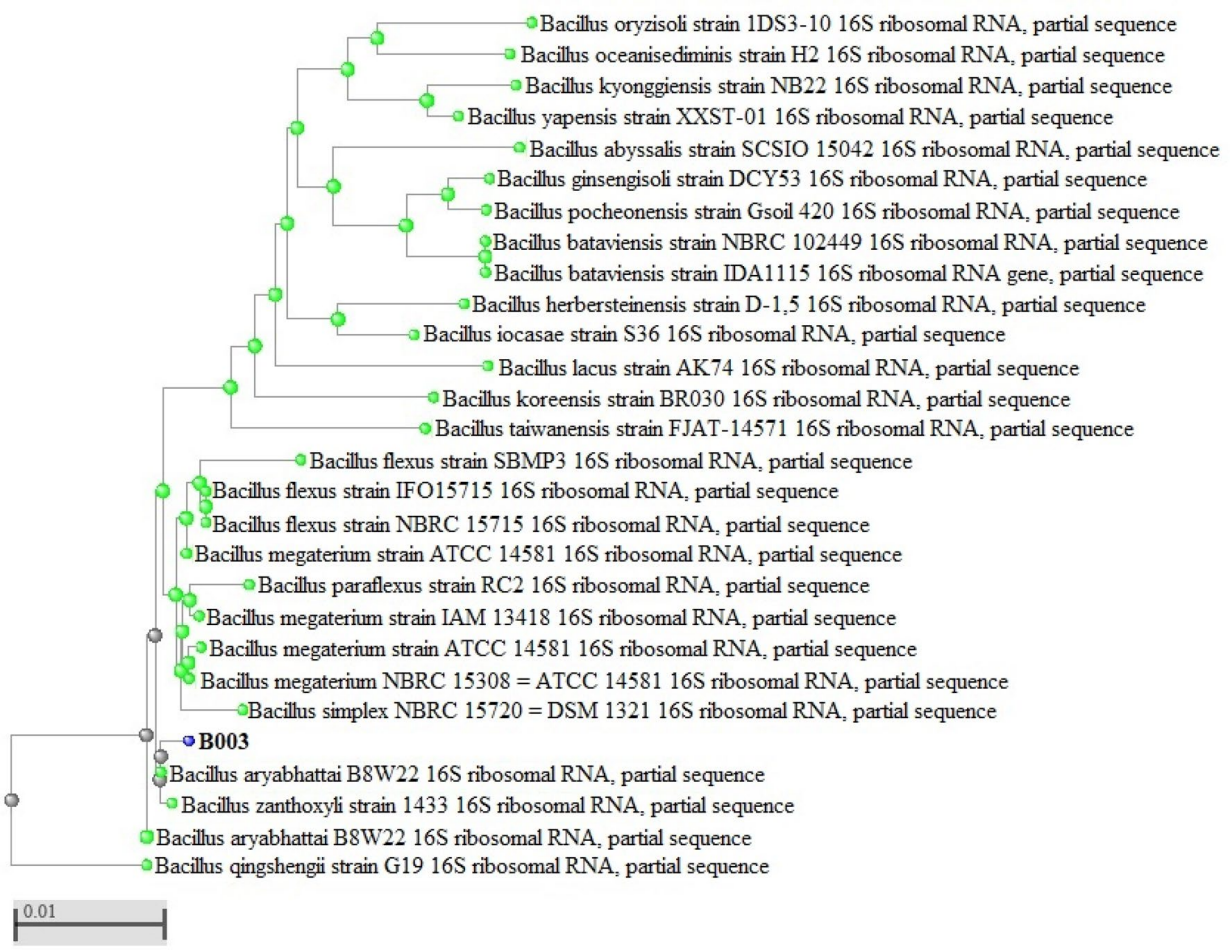
Phylogenetic tree of 16S rRNA sequences from the endophytic bacterium strain (B003) compared with other representative members of the *Bacillus* genus.

One strain (B003) was isolated from the wild plant species at a concentration of 3.1 × 10^2^ cfu/cm of fresh root. Strain B003 was a small, rod-shaped, gram-positive, spore-forming bacterium belonging to the genus *Bacillus*. The bacterial strain was identified using the partial (1147 bp) 16S rRNA gene. Using the taxonomically united database of 16S rRNA in EzBioCloud, 16S rRNA was identified as a top hit with *Bacillus aryabhattai* with 100% similarity. Considering the identification results, a phylogenetic tree was constructed by comparing the 16S rRNA gene sequences of strain B003 with the reference strain sequences from the National Center for Biotechnology Information (NCBI) GenBank public database. Molecular analysis indicated that the isolated strain B003 belongs to the genus *Bacillus*, with an identity percentage of 100% and an E-value of 0.0 with *B. aryabhattai* (Fig. 1).

### Induction of defense-related genes in *Nicotiana tabacum* by culture filtrate, total protein and *B. aryabhattai* HKEB

**Figure 2:**
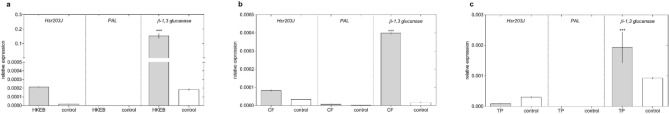
Relative level of expression of several genes associated with plant disease resistance in *N. tabacum* plants that were treated with HKEB (**a**), culture filtrate (CF) (**b**), and total protein (TP) (**c**) from *B. aryabhattai*. A molecular marker of the hypersensitive response (Hsr203J), PAL and β-1,3 glucanase were used in the analysis. The bars show the mean values with standard errors (n = 5).

To evaluate the effects of HKEB, culture filtrate and total proteins from *B. aryabhattai* on plant defense, we analyzed the expression profiles of several genes involved in plant defense in *N. tabacum* plants. Plants that were treated with HKEBs (Fig. 2a) and culture filtrate (Fig. 2b) showed significant expression of the β-1,3 glucanase gene, which was induced 1000-fold and 20-fold relative to that in controls, respectively. Total proteins isolated from *B. aryabhattai* (Fig. 2c) also induced the expression of the β-1,3 glucanase gene but to a lesser extent (twofold) relative to that in the control. Expression of the *Hsr203J* gene was also detected in plants that were treated with HKEBs (Fig. 2a) and culture filtrate (Fig. 2b). However, the expression levels were not significantly different from that in the control. Additionally, the detected expression of phenylalanine ammonia-lyase (*PAL)* after treatment with culture filtrate (Fig. 2b) of endophytic bacteria was low.

### Induction of defense-related genes in *A. thaliana* by *B. aryabhattai* HKEB

**Figure 3:**
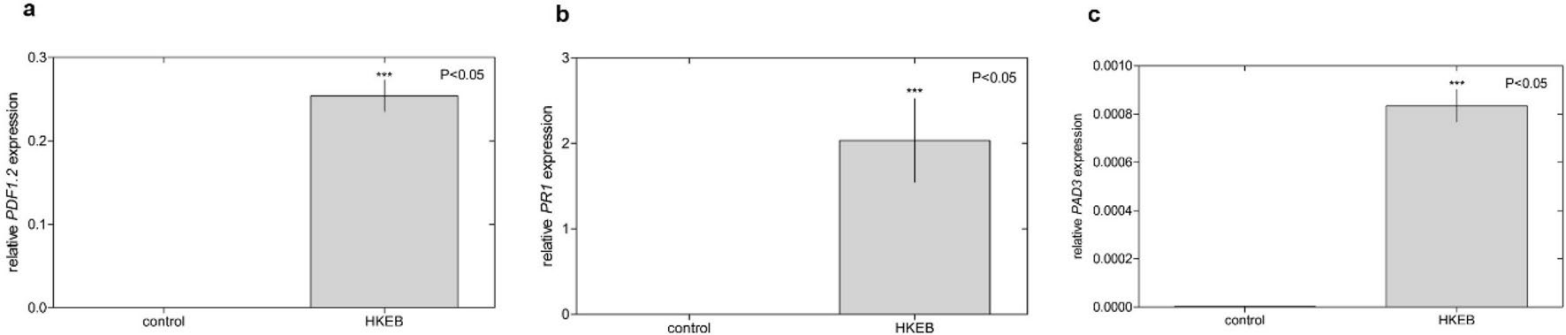
*B. aryabhattai* HKEB induced high defense gene expression in Arabidopsis plants. The relative expression of the *PDF1.2* (**a**), *PR1* (**b**) and *PAD3* (**c**) genes involved in plant defense was evaluated in Arabidopsis. The bars represent the mean values and standard errors of the means (n = 9). The experiments were replicated three times.

To detect the induction of defense genes in Arabidopsis plants treated with HKEB, we evaluated the expression of plant defensin 1.2 (*PDF1.2*), pathogenesis-related protein 1 (*PR1*) and phytoalexin deficient 3 (*PAD3*). *B. aryabhattai* HKEB induced significant expression of all the defense genes evaluated in Arabidopsis plants. The relative expression of *PAD3* was higher than that of the other evaluated genes, showing approximately 82-fold induction (Fig. 3c). While the HKEBs increased the expression level of *PDF1.2* 25-fold (Fig. 3a), *PR1* gene expression was 20-fold higher in treated plants than in the control (Fig. 3b).

**Figure 4:**
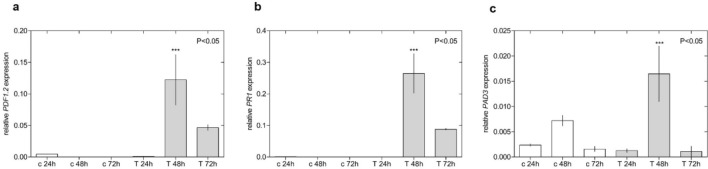
Time course of gene expression in Arabidopsis plants treated *B. aryabhattai* HKEB. The *PDF1.2* (**a**), *PR1* (**b**) and *PAD3* (**c**) genes involved in plant defense were evaluated in Arabidopsis. The bars represent the mean values and standard errors of the means (n = 9). The experiments were replicated three times.

The evaluation of gene expression in Arabidopsis plants treated with HKEB at different time points revealed that all the genes (*PDF1.2*, *PR1 *and* PAD3*) showed the same behavior with respect to time (Fig. 4). Significant induction was detected 48 h after treatment. A higher level of relative gene expression induction (120-fold) was recorded for *PDF1.2* than for the other genes (Fig. 4a). While the expression of *PR1* gene was induced 27-fold (Fig. 4b), the lowest induction level was observed for *PAD3* gene expression (2.42-fold) relative to the expression in control plants (Fig. 4c). Gene expression was reduced approximately 2.5-fold after this time for all cases.

### HKEB induce an effective defense in *N. tabacum* and *A. thaliana* against pathogens

**Figure 5:**
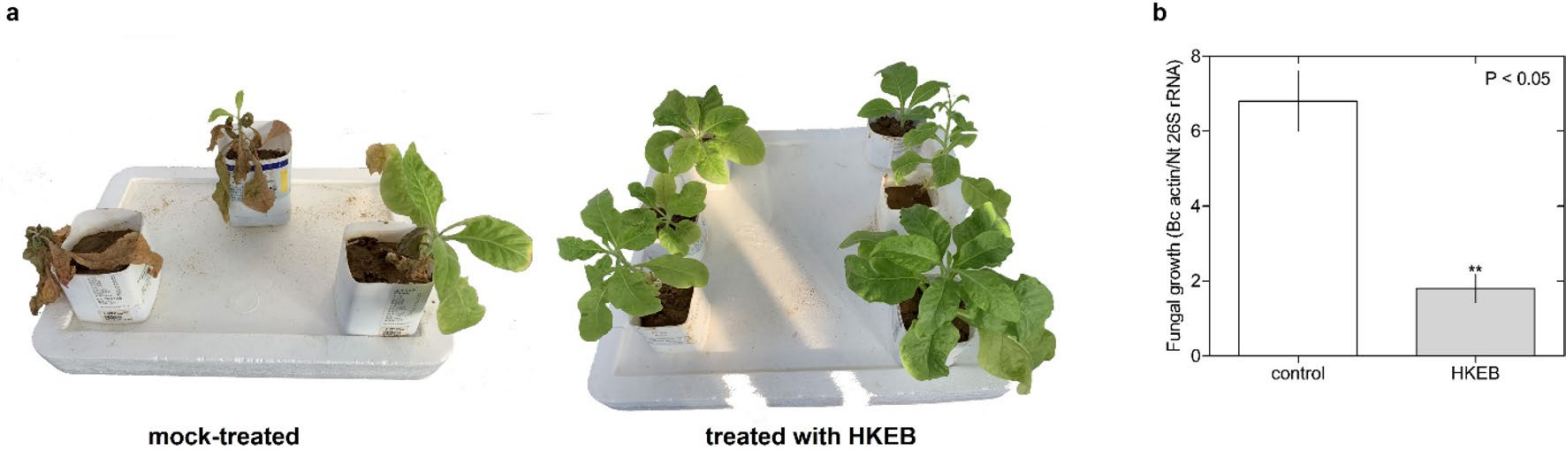
*B. aryabhattai* HKEB induced an effective defense against *B. cinerea* infection. (**a**) Disease symptom evaluation in tobacco plants that were mock-treated and treated with HKEB 1 week post-inoculation. (**b**) *In planta* fungal growth was evaluated using the relative expression of the *B. cinerea* β-actin gene. The bars represent the mean values and standard errors of the means (n = 10). The experiments were replicated three times.

To test whether the application of *B. aryabhattai* HKEB induced protection against the necrotrophic pathogen *B. cinerea*, we inoculated *N. tabacum* plants with the pathogen and started the application with HKEB 24 h post-inoculation. Figure 5a shows the phenotypes of plants treated with HKEB and water. There was effective protection in *N. tabacum* plants treated with HKEB compared with plants treated with water, and severe symptoms of *B. cinerea* infection were observed in the plants that were treated with water. In addition, *B. cinerea* growth was higher (3.7-fold) in the control plants than in the plants treated with HKEB, based on quantification of actin gene expression (Fig. 5b).

**Figure 6:**
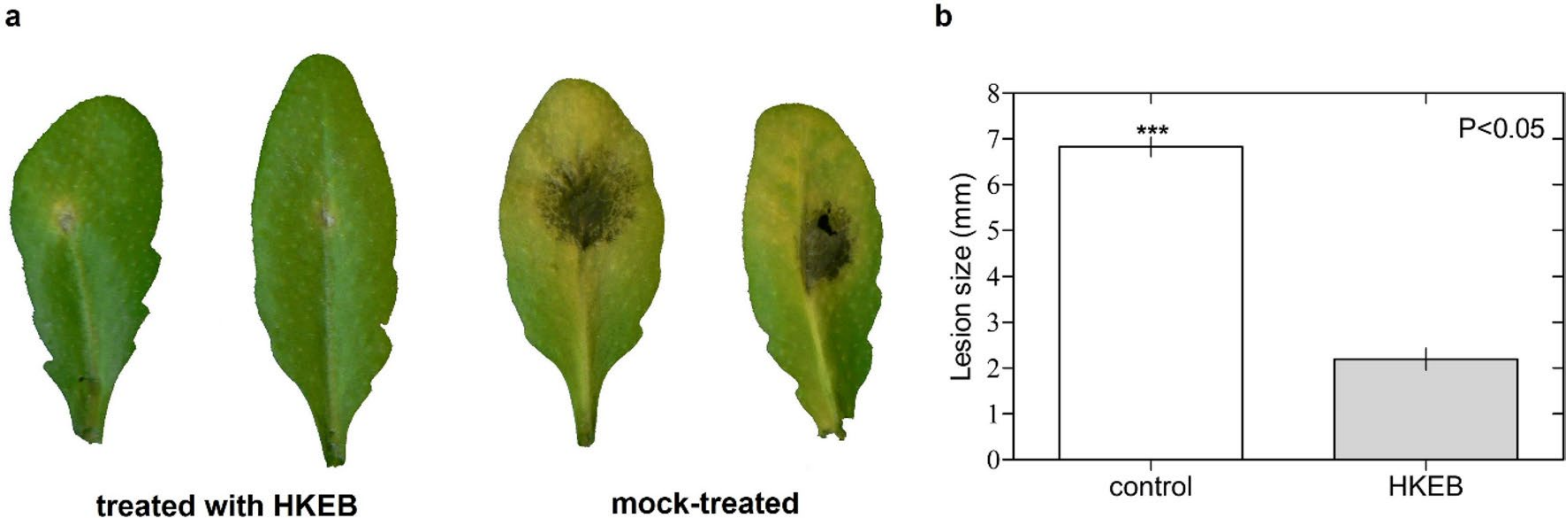
*B. aryabhattai* HKEB induced an effective defense against *B. cinerea* infection in Arabidopsis plants. (**a**) Disease symptom evaluation in Arabidopsis plants that were treated with HKEB and mock-treated at 72 h post inoculation. (**b**) The lesion size produced by *B. cinerea*. The bars represent the mean values and standard errors of the means (n = 10). The experiments were replicated three times.

To analyze whether the application of *B. aryabhattai* HKEB also protects Arabidopsis plants against *B. cinerea*, we evaluated the symptoms and lesion size produced by the pathogen in the plants that were treated with HKEB and water. *B. cinerea* produced severe spots in the control plants compared with the plants treated with HKEB (Fig. 6a). Moreover, lesion size was significantly larger in the plants that were treated with water (6.8 mm) than in the plants that were treated with HKEB (2.1 mm) (Fig. 6b).

**Figure 7:**
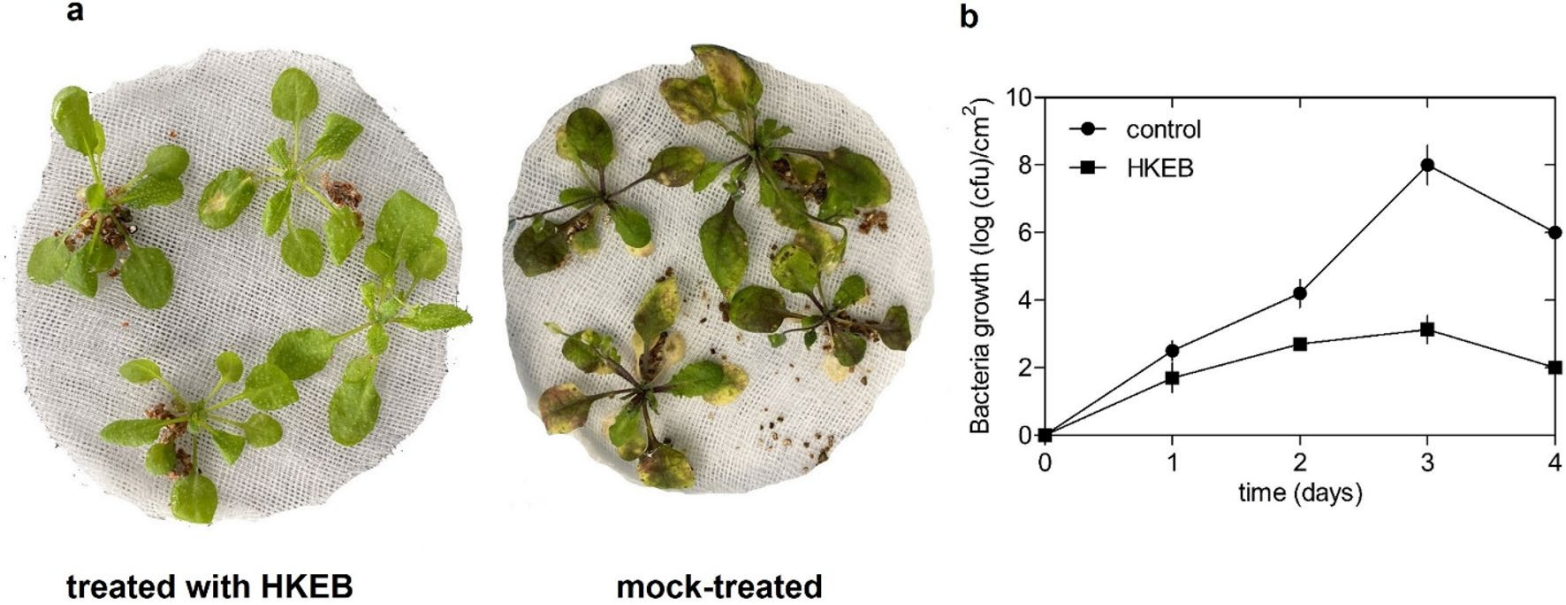
HKEB from *B. aryabhattai* induce an effective defense against *P. syringae* pv. *tomato* DC3000 strains in Arabidopsis plants. (**a**) Disease symptom evaluation in Arabidopsis plants that were treated with HKEB and mock-treated 1 week post-inoculation. (**b**) Bacterial growth in Arabidopsis plants. The multiplication of *Pst* in Arabidopsis leaves was plotted on a log scale. The bars represent the mean values and standard errors of the means (n = 10). The experiments were replicated three times.

Furthermore, we evaluated the protective effect of HKEB against the biotrophic pathogen *P. syringae* pv. tomato DC3000 (*Pst*) strain. Most Arabidopsis plants that were treated with HKEBs (1.8 log (cfu)/cm^2^) showed a reduction in symptoms compared with those of the plants that were treated with water (5.9 log (cfu)/cm^2^) after 4 days (Fig. 7a). Foliar application of HKEBs significantly reduced the accumulation of the *Pst* strain (Fig. 7b).

### RNA sequencing-based detection of new defense-related genes in *A. thaliana* treated with HKEB

**Figure 8:**
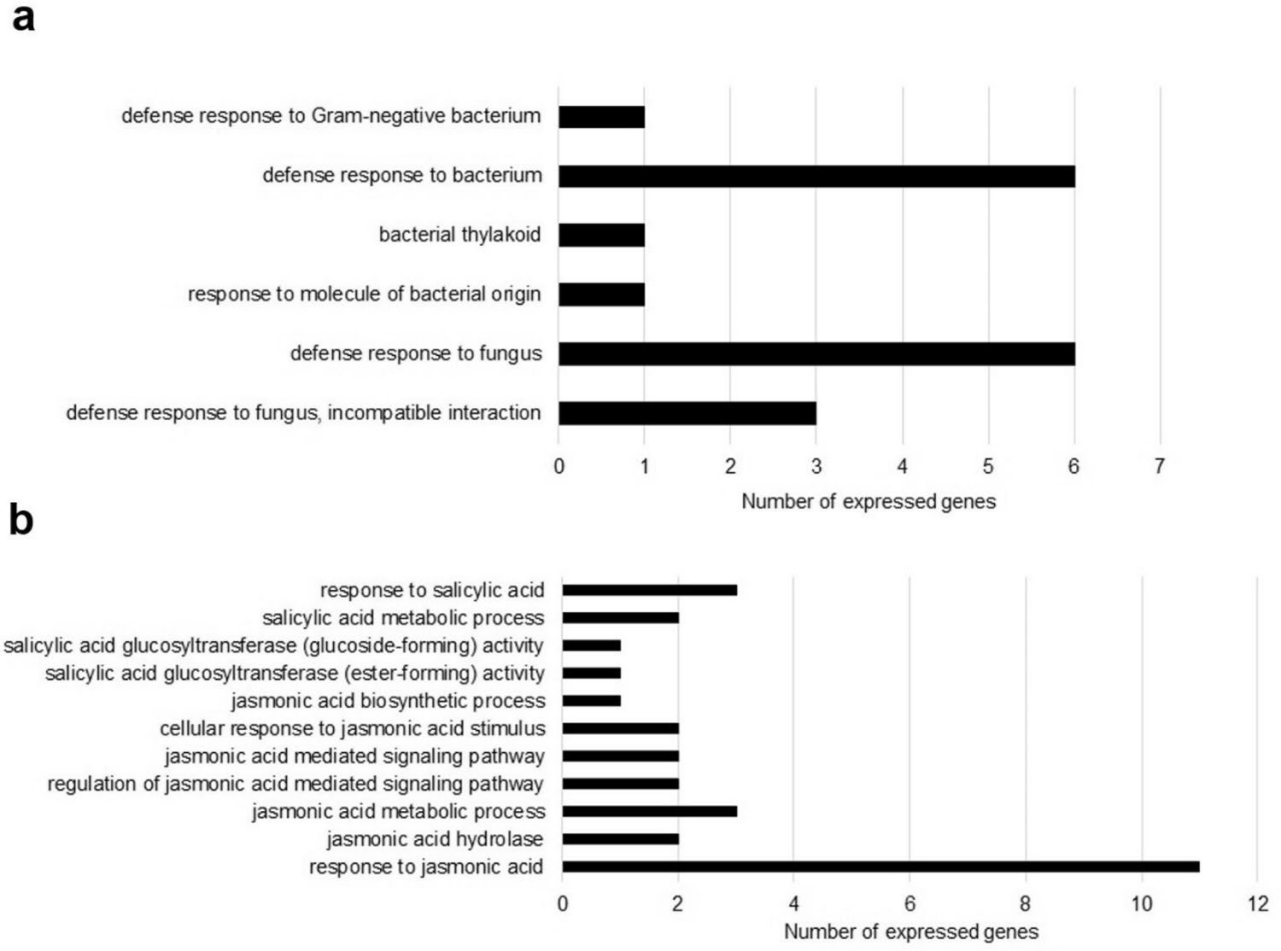
RNA sequencing analysis of differentially expressed genes in Arabidopsis plants that were treated with *B. aryabhattai* HKEB. (**a**) Number of expressed genes related to plant defense against pathogens. (**b**) Number of expressed genes related to the JA and SA signaling pathways.

To identify new genes induced by *B. aryabhattai* HKEB treatment in Arabidopsis plants, a transcript profile analysis was conducted using RNA sequencing. Arabidopsis plants that were treated with HKEB and water were collected 72 h after foliar application. Although RNA sequencing produces a large quantity of information, we focused our analysis on the number of significantly expressed genes related to general plant defense and salicylic acid (SA)/JA signaling pathways. In this analysis, the genes that were related to the defense response to bacteria were the most represented, with 132 expressed genes, followed by the genes involved in the defense against fungi, with 118 genes (Fig. 8a). The number of expressed genes related to the JA pathway was more representative than that related to the SA pathway, with 11 expressed genes (Fig. 8b) (Supplementary Table S1).

### Analysis of defense responses triggered by HKEB treatment in Arabidopsis mutants

**Figure 9:**
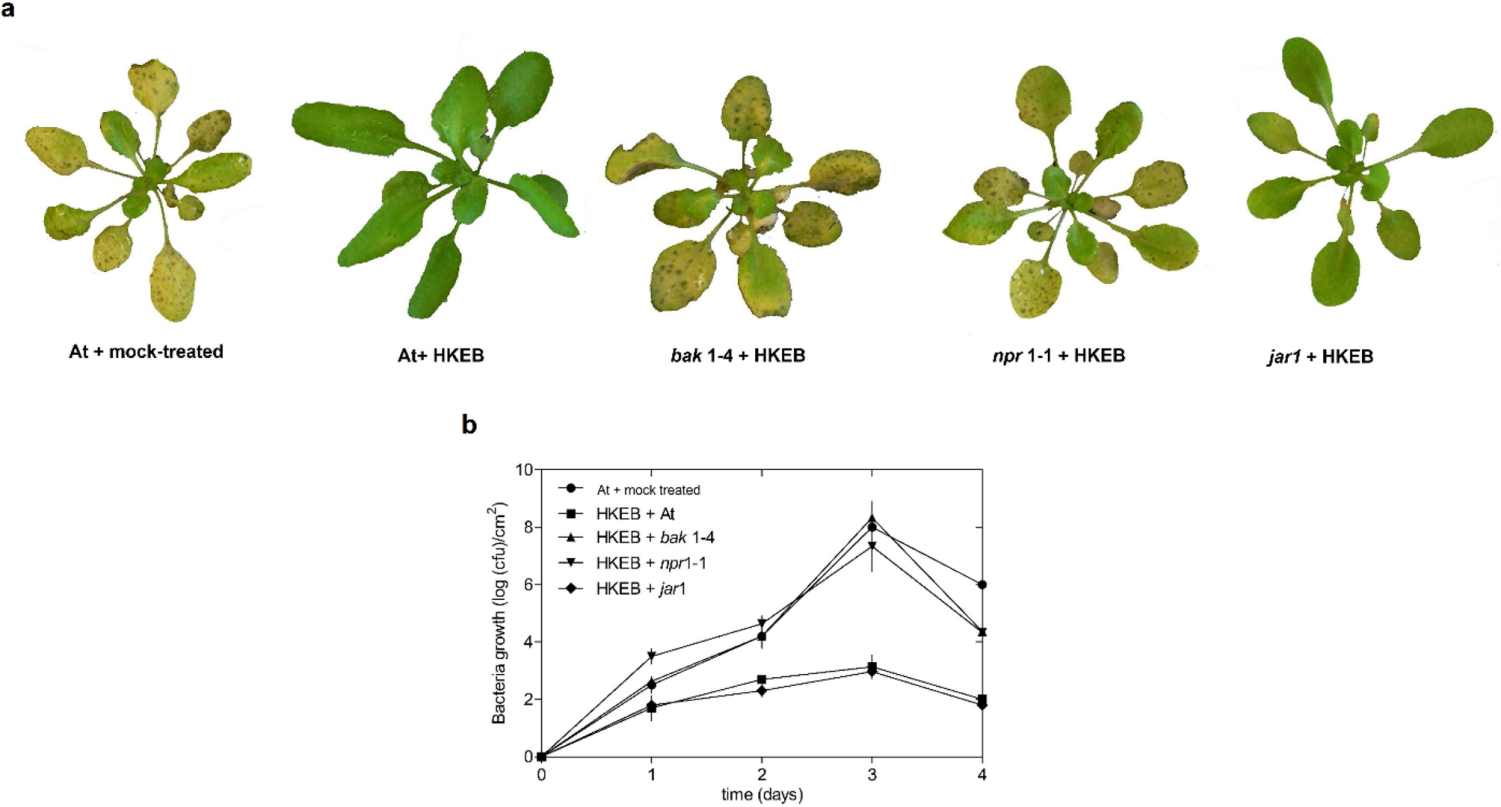
The defense responses that are triggered by *B. aryabhattai* HKEB treatment are dependent on BAK1 and the SA pathway against the biotrophic pathogen *Pst*. (**a**) Phenotypes of bak1-4, npr1-1 and jar1 mutant plants that were treated with HKEB. (**b**) Bacterial growth in mutant plants that were treated with HKEB. The *in planta* bacterial populations were determined daily. The multiplication of *Pst* in Arabidopsis leaves was plotted on a log scale. The bars represent the mean values and standard errors of the means (n = 10). The experiments were replicated three times.

**Figure 10:**
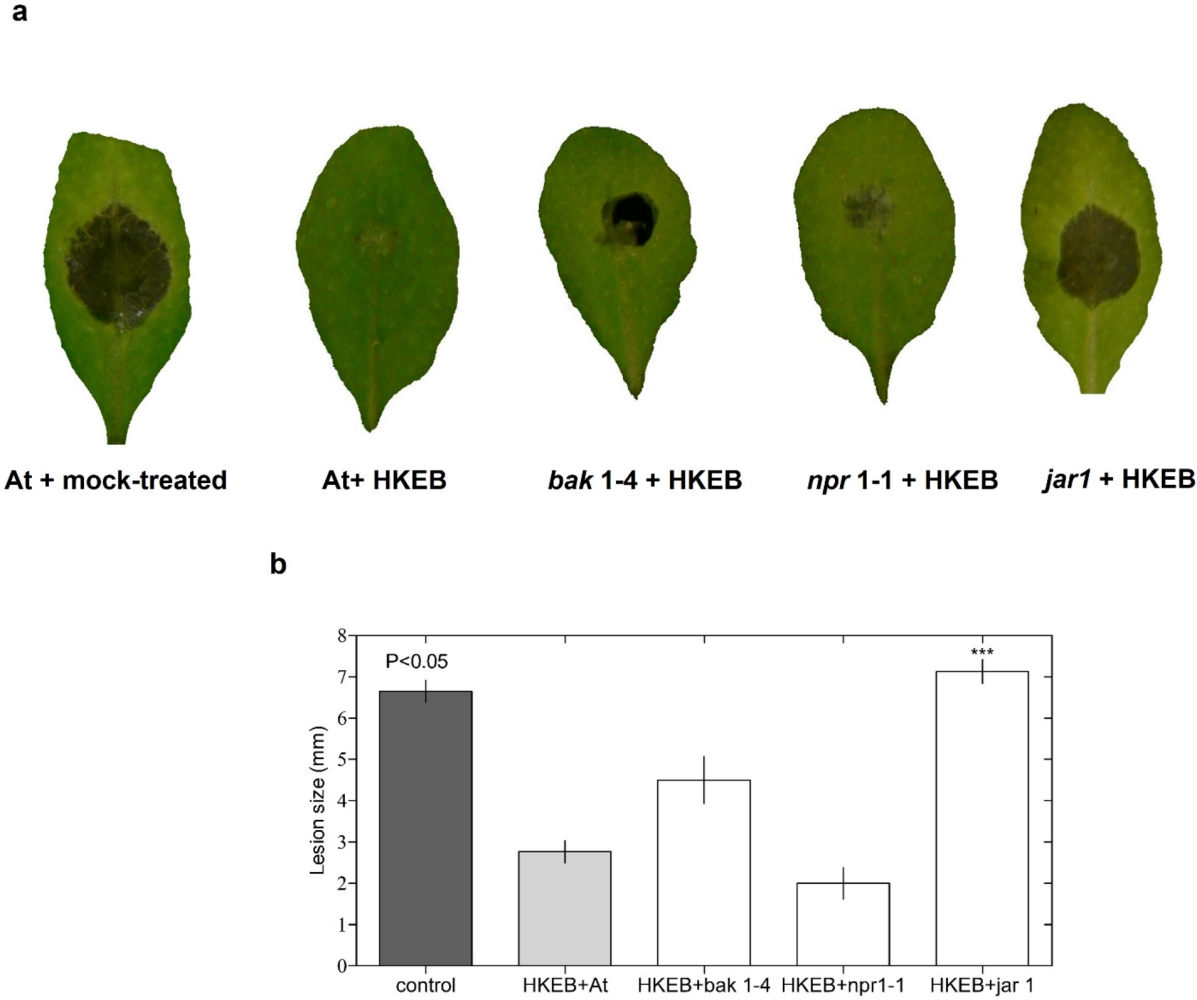
The defense responses that were triggered by *B. aryabhattai* HKEB treatment are dependent on the JA pathway against the necrotrophic pathogen *B. cinerea*. (**a**) Disease symptom evaluation in Arabidopsis mutant plants that were treated with HKEB at 72 h post-inoculation. (**b**) The lesion size produced by *B. cinerea* was determined. The bars represent the mean values and standard errors of the means (n = 10). The experiments were replicated three times.

Using Arabidopsis mutants (*bak1*, *npr1* and *jar1*), we characterized the defense responses triggered by HKEB treatment against *P. syringae* pv. tomato DC3000 and *B. cinerea*. The *bak1* and *npr1* mutants of Arabidopsis showed compromised defense responses against *P. syringae* pv. tomato DC3000 when treated with HKEB. Typical disease symptoms and bacterial growth were observed (Fig. 9). Mutation of the *jar1* gene did not compromise the defense reaction (Fig. 9). In contrast, mutation of the *jar1* and *bak1* genes compromised the resistance of Arabidopsis mutant plants treated with HKEB against *B. cinerea* (Fig. 10)*.* However, the *bak1* mutant plants showed a smaller lesion size than the control plants (Fig. 10).

### Biochemistry characterization of the HKEB

**Figure 11:**
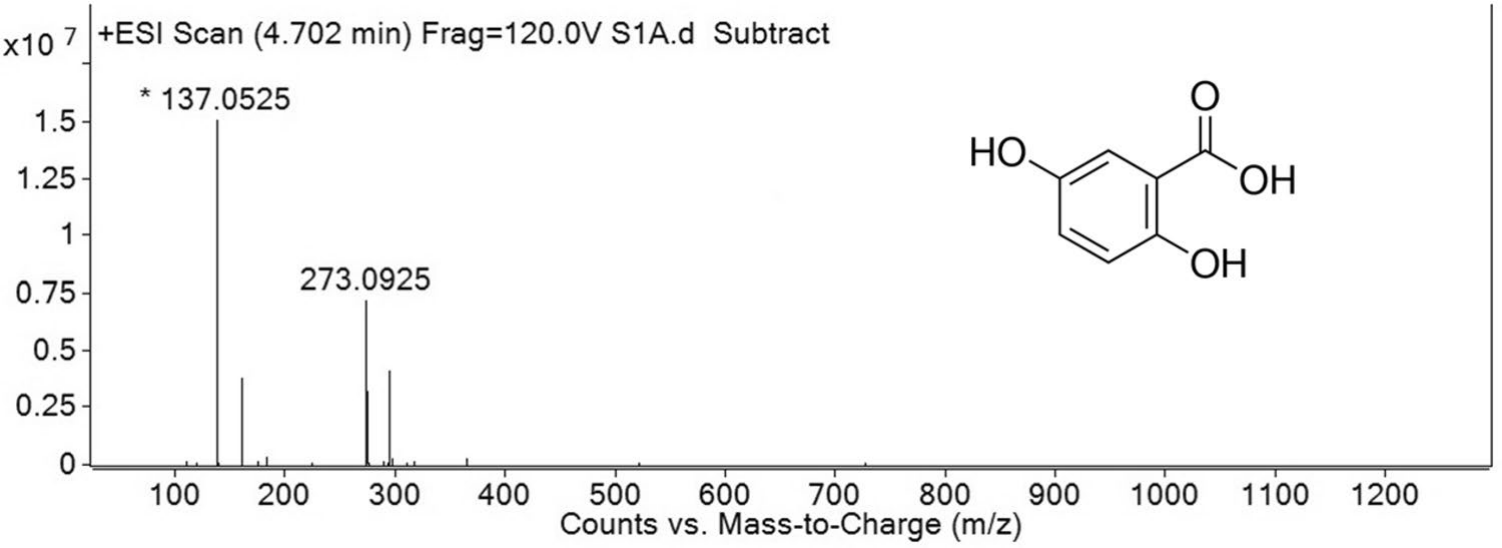
HPLC–MS spectrum of gentisic acid found in *B. aryabhattai* HKEB.

High-performance liquid chromatography (HPLC)/mass spectrometry (MS) enabled the biochemical characterization and identification of the different molecules present in the *B. aryabhattai* HKEB, and gentisic acid was identified in the HKEB preparation (Fig. 11). Moreover, lipoteichoic acid, peptidoglycans and exopolysaccharides were identified (Supplementary Table S2).

## Discussion

The soils of the numerous ecosystems on Earth host different groups of bacteria. Plants contain a significant number of endophytic microorganisms, which play an important role in plant life and perform critical support functions.

An endophytic bacterium was isolated from a wild plant species with stable and consistent growth. The phylogenetic analysis showed that the endophytic bacterial strain belongs to the genus *Bacillus* and highly similar to *B. aryabhattai* in terms of 16S rRNA nuclear sequence (Fig. 1). Recent studies have shown that endophytic microorganisms, including some species of the genus *Bacillus,* play an important role in plant defense^48^. These endophytic microorganisms often suppress plant pathogens. A study showed that the endophytic microbiome triggered innate plant defense against the root pathogen *Rhizoctonia solani* and explained how this suppression mechanism operated at the second microbiological level of plant defense^49^. For example, a *Bacillus xiamenensis* strain was involved in the control of different sugarcane pathogens. In vitro and in vivo assays showed that *B. xiamenensis* developed strong antagonistic activity against important fungal pathogens. In addition, some antioxidative enzymes are produced and possibly involved in the activation of sugarcane plant defense^50^.

Inducing a natural defense against diseases is currently of great importance and interest, as it allows the molecular and biochemical systems that are already present in the plant to be used for disease control. The full arsenal of plant responses to diseases includes different actions involved in recognition, signaling and response, which are defined as plant innate immunity^6^. Innate immunity may be induced by diverse elements that provide disease control. The synthesis of compounds, such as phytoalexins, defensins and pathogenesis-related (PR) proteins, are defense mechanisms that are triggered “*in planta”*. Most of these responses are mediated by the activation of genes related to SA, JA/ethylene (ET) and hypersensitive responses^6^.

Interestingly, HKEB, culture filtrates and total proteins from *B. aryabhattai* induced high defense gene expression in *N. tabacum* plants. Notably, the expression of the β-1,3 glucanase gene was 1000-fold induced by HKEB (Fig. 2). Plant β-1,3-glucanases have been classified as the PR-2 family of pathogenesis-related proteins^51^. Previous results have shown that upregulation of the PR-2 gene was associated with SAR during pathogen infection or following exogenous application of SA^52^. Gentisic acid (2,5-dihydroxybenzoic acid), a main compound recovered in the HKEB fraction (Fig. 11), is a metabolic derivative of SA^53^ and could be responsible for the strong induction recorded for the β-1,3-glucanase gene.

The Hsr203j and PAL genes were not expressed when the total proteins from endophytic bacteria were used. It is likely that such proteinaceous compounds were not recognized by the receptors that trigger the expression of these genes (Fig. 2). However, the HKEB and secondary metabolites that were segregated in the culture filtrate induced the expression of the Hsr203j gene approximately 20- and 3-fold relative to that in the controls, respectively. This gene is associated with a hypersensitive response in tobacco plants^54^. Moreover, the PAL gene was induced (2.8-fold) only when the culture filtrate was used. This is a key gene both in the phenylpropanoid pathway and for the enzyme that produces precursors of several secondary metabolites (phytoalexins) involved in plant defense^55^.

PAMPs and MAMPs are molecules produced by microorganisms and perceived by receptor molecules in the plant that activate defense signaling pathways and limit pathogen invasion^10^. These molecules are conserved in bacteria and induce different types of plant defenses. In the current study, we showed that the application of *B. aryabhattai* HKEB in *A. thaliana* plants stimulated plant defense pathways through the induction profile of the genes involved in plant defenses. The expression of the *PR1* and *PDF1.2* genes associated with the SA and JA pathways was similarly induced in Arabidopsis plants treated with *B. aryabhattai* HKEB (Fig. 3)*.* The expression of the *PAD3* gene, which is generally related to the SA pathway, was strongly induced relative to that in control plants. The expression of these defense genes in Arabidopsis plants reached their maximum level 48 h after the application of HKEBs, after which the expression was significantly reduced (Fig. 4). Interestingly, the RNA sequencing data showed that most of the genes that were expressed were associated with the JA pathway.

Several signaling elements that are induced by pathogens include SA, JA, and ET. SA-and ET/JA-mediated signaling pathways play important roles in plant resistance against pathogens^56^. The SA signaling pathway controls plant defense mechanisms against biotrophic pathogens, whereas the ET/JA pathways are usually required for plant resistance to necrotrophic pathogens^57,58^. JA, ET and SA signaling are required for endophyte-mediated resistance. Endophytes can activate the SA and JA signaling pathways^59,60^. Generally, the JA and ET pathways induce resistance against necrotrophic pathogens, whereas the SA pathway triggers resistance against biotrophic and hemibiotrophic pathogens^56^.

SA is an important plant hormone that mediates host responses to pathogen infection^61^. SA content is a signal that increases in response to pathogens^61,62^, and this increase is related to the induction of antimicrobial PR genes to enhance disease resistance^61,63,64^. On the other hand, *PAD3* mutation results in a drastic reduction in camalexin production. Camalexin is involved in resistance to fungal pathogens^65^. SA is important for camalexin synthesis and *PR-1* expression^66,67^, and SA application induces plant resistance to several pathogens^68^. In addition, some experiments have shown that the jasmonic acid signaling pathways must be triggered to induce *the PDF1.2* gene after pathogen infection in Arabidopsis plants^69^.

Furthermore, the effect of treatment with HKEB on the defense responses of *N. tabacum* and Arabidopsis plants against pathogens was evaluated. The application induced resistance in *N. tabacum* (Fig. 5) and Arabidopsis plants against fungal (Fig. 6) and bacterial (Fig. 7) pathogens, and HKEB elicited natural defense in these two plant species. No symptoms were observed in the *N. tabacum* and Arabidopsis plants that were inoculated with two different pathogens and treated with HKEB. Our results showed that *N. tabacum* and *A. thaliana* plants activated strong defense mechanisms against the *B. cinerea* and *Pst* pathogens, respectively. These results showed the induction of plant defense by *B. aryabhattai* HKEB against necrotrophic and biotrophic pathogens. The results showed that although a plant might react in the presence of HKEB, the defense response must be fully mobilized to provide complete protection. Therefore, 24 h might be required.

Our analysis showed that using HKEB and defense-compromised mutants of *A. thaliana* yielded resistance to *Pst* via the *bak1* and *npr1* genes, whereas *jar1* was not essential (Fig. 9). On the other hand, the results with the defense-compromised mutants showed that resistance to *B. cinerea* mediated by HKEB occurs primarily through the *jar1* gene pathway and requires JA components (Fig. 10). Although *bak1* plants treated with HKEB were affected by the pathogen, the effect was weaker than that in the *jar1* mutant plants.

In general, bacteria can produce diverse molecules that are capable of inducing plant defense against pathogens. For example, peptidoglycan elicited defense in *A. thaliana*, rice and tobacco plants^19,70–72^, and biosurfactants, such as rhamnolipids and lipopeptides, which are produced by *Pseudomonas* and *Bacillus,* are also capable of activating systemic resistance^73^.

Interestingly, we detected traces of gentisic acid, which is a derivative of SA, in *B. aryabhattai* HKEB (Fig. 11). In addition to SA content, gentisic acid content is a signal during the induction of plant defense against necrotic pathogens^74^. Furthermore, treatments with gentisic acid triggered resistance to RNA pathogens in tomatoes and *Gynura auriantiaca*^75^. SA is produced by endophytic bacteria that induce systemic resistance^76^. A previous study showed that the *Burkholderia* sp. strain BC1, which is a soil bacterium, produces SA and gentisic acid^77^.

Our data support the idea that once a bacterium is inactivated, mainly through heat treatment, dead cells may release bacterial components with important immunomodulatory effects against pathogens. Bacterial components, such as exopolysaccharides, peptidoglycans and lipoteichoic acids, are involved in these properties in preparations containing heat-killed bacteria^78,79^. These results show that heat-killed bacteria or their fractions are a key trigger for the activation of plant defense. Importantly, this is the first report that gentisic acid produced by *Bacillus* species could be involved in the activation of plant defense. Heat-killed bacteria have shown immunomodulatory functions in different experiments using animals and humans. The effect has been evaluated in the treatment of different diseases^27,28^. Most of the mechanisms used by heat-killed bacteria to induce programmed cell death in animals are homologous to the plant hypersensitive response, which is a type of localized programmed cell death^80^.

In summary, our results strongly suggest that HKEB-induced defense restricts pathogen multiplication and disease development. The use of HKEBs might be an option for defense activation in the control of plant diseases using a mixture of different MAMPs and heat-killed bacteria alongside a suitable delivery system.

## Methods

### Isolation of the endophytic bacterium

Samples were collected from the wild plant species *G. chinensis* (Keng) along the Fu Tuan River (35° 20′ 17″ N, 119° 26 ′8″ E) within 5 km^2^ of the coastal region of Rizhao city in Shandong Province, People's Republic of China. *G. chinensis* (Keng) was identified according to the data on morphological traits from the Flora of China (http://www.iplant.cn/foc/). This experimental study complies with Chinese national and local laws, and sample collection was permitted by the Rizhao Administration and Municipal Sciences and Technology Department. (Collection information: South China Botanical Garden (IBSC) of the Chinese Academy of Sciences. Source: China Digital Plant Specimens Museum. Identifier: 0114164. Collector: Zhang Zhisong Acquisition number: 401467). A total of 100 samples were randomly collected during spring. First, the plant material (stems and roots) was rinsed with water. The samples were then sliced using a sterilized blade under aseptic conditions. Each sample was surface sterilized with 70% ethanol for 1 min and then immersed in a sodium hypochlorite solution (5%) for 1 min. The samples were treated with sterile distilled water for 1 min and dried on filter paper. After proper drying, pieces of plant parts were placed in 1 ml of sterile water and physically treated in a TissueLyser (Qiagen, Hilden, Germany) for 5 min. The debris was decanted, and 100 µl of the remaining water was incubated in Luria–Bertani (LB) agar medium (yeast extract, 5 g/l: peptone, 10 g/l; sodium chloride, 5 g/l; agar, 12 g/l; pH 7) at 37 °C for 3 days. Parallel to the samples, the final wash solution from the surface sterilization procedure was also spread plated onto the MS medium, which served as a control. The bacteria were isolated only from internally processed samples. This was the criterion used to classify them as endophytes and not surface contaminates. Bacterial colonies were selected based on growth rate, colony morphology and pigmentation. Colony morphology was described on the basis of size, shape, texture, elevation, pigmentation, and growth medium effect. The features included shape (circular, irregular, or punctiform), margin entirety (smooth with no irregularities), elevation, texture (mucoid, moist-wet) or pigment color (colorless, white, or off-white; no diffusible pigment; diffusible/water soluble pigments). Additionally, individual bacterial populations with the highest cfu/cm fresh root were harvested. Bacterial isolates were selected and purified by a streaking procedure. These isolates were incubated at 37 °C. Pure cultures of the bacterial strains were maintained in 30% glycerol at − 80 °C.

### Plant materials and growth conditions

*Nicotiana tabacum, A. thaliana* Col-0 and Arabidopsis mutant plants (*bak1-4*, *npr1-1*, and *jar1*) were used in the experiments. Surface-sterilized seeds were plated on Murashige and Skoog (0.5X MS) basal media (Sigma, St Louis, MO, USA) with 1% w/v sucrose at 4 °C in the dark for 2 days and placed in a controlled growth room at 220 °C with a photoperiod of 16 h of light/8 h of dark. Small Arabidopsis plants were transferred to a mixture of soil composed of peat plugs and vermiculite in a 1:1 ratio for 14 days. *N. tabacum* plants were grown in six-inch pots containing black turf and rice husk (4:1) and kept in growth chambers at 23 °C with a photoperiod of 16 h of light/8 h of dark.

### Pathogen inoculation procedure

The *B. cinerea* strain was grown on V8 medium agar for 15 days at 24 °C before spore collection. Leaves of 4-week-old *A. thaliana* and tobacco plants were inoculated (10 μl of spore suspension placed on top of the leaf) at a density of 500,000 conidia/ml after being germinated in a 12 g/l potato dextrose broth at room temperature for 3 h^81^. Furthermore, disease assays were performed on whole plants by spraying the spore suspension mentioned previously. Inoculated plants were grown under a transparent cover to obtain high humidity. The final evaluation was performed 3 days later. The lesion diameter and symptoms were measured 3 days post-inoculation^81^.

Meanwhile, the *P. syringae* pv. tomato DC3000 strain was grown in King’s B medium with 50 μg/ml rifampicin overnight at 28 °C^82^. The bacteria were diluted to the desired density using water. Leaves from the 4-week-old plants were sprayed with *Pst* at a concentration of 5 × 10^8^ cfu/ml in water with 0.02% Silwet L-77^71^. Bacterial counting was performed on seven leaves with three replicates by surface sterilization with 70% ethanol 3 days post-inoculation^82^.

### Production of culture filtrates, total proteins and HKEBs

The isolated bacterial strain was incubated in 100 ml of LB broth in a 250-ml Erlenmeyer flask with shaking (200 rpm) for 2 days at 37 °C in the dark. Fermentation with an optical density of 2.1 was used to extract the culture filtrate and total proteins from the bacterial strain. Culture filtrate of the bacterial strain was obtained by centrifugation at 8000×*g* for 10 min and filtered (0.22 μm, Millipore). Total protein from the bacterial strain was extracted using a Total Protein Extraction Kit (Sangon Biotech, Shanghai, CHINA). The HKEBs were obtained physically, as the bacterial pellets were treated three times for 30 s in liquid nitrogen, 30 s in hot water and 10 min in a TissueLyser (Qiagen). The HKEBs were washed twice with sterile water and dried. The HKEBs (100 mg of dried solid) were diluted in 10 ml of a solution of water and ethanol (5:1). The composition in a final volume of 100 ml during the spray application was as follows: 1—culture filtrate: 10 ml of culture filtrate + 90 ml of sterile water; 2—total protein: 1 ml of total protein + 99 ml of sterile water; and 3—10 ml of HKEBs [dissolved in water:methanol (5:1)] + 90 ml of sterile water. Water and mock treatment (10 ml of solution without HKEBs (water:methanol (5:1) + 90 ml of sterile water) were used as controls. The culture filtrates and total protein were used to evaluate the expression of genes involved in *N. tabacum* plant defense against diseases. The HKEBs were used to evaluate the expression of genes involved in *N. tabacum* and *A. thaliana* plant defense against diseases. Furthermore, the HKEBs were used in the evaluation of defense responses against pathogens.

### Identification of the endophytic bacterium

The selected bacterial strain was grown in LB broth, and DNA was extracted according to the protocol described by Sambrook et al*.*^83^. For molecular identification, a 16S rRNA gene sequence was amplified by polymerase chain reaction (PCR) using the 27F and 1492R primers listed in Table 1. Amplification was conducted in a Thermal Cycler T100 machine (Bio-Rad, Shanghai, China) using a Taq PCR Master Mix Kit (Qiagen). The PCR cycles were as follows: 95 °C for 10 min; 35 cycles of 95 °C for 30 s, 55 °C for 10 min and 72 °C for 1.5 min; and a final extension at 72 °C for 10 min. The PCR fragment was sequenced using an ABI 3730 DNA sequencer (Applied Biosystems, CA, USA). The 16S rRNA gene fragment sequence (1147 bp) was identified using BLASTN homology searches^84^. The examined databases included the National Center for Biotechnology Information (NCBI)-GenBank, European Molecular Biology Laboratory-European Bioinformatics Institute (EMBL-EBI) and EzBioCloud taxonomically united 16S rRNA (https://www.ezbiocloud.net) databases^85^. CLUSTAL Omega software was used for sequence alignment^86^. The neighbor-joining method was used to construct the phylogenetic tree^87^. The assigned 16S rRNA gene sequence accession number was MW899048.

**Table 1:** Oligonucleotides used in the experiments.

Genes analyzed	Oligonucleotides
*At* PAD3: glutathione S transferase	5′-TTGGCTTCTGACCACTTCAC-3′ 5′-ACGCTCGTCGAAGAGTTTCT-3′
*At* PR1: pathogenesis-related protein	5′-GATGTGCCAAAGTGAGGTG-3′ 5′-CTGATACATATACACGTCC-3′
*At* PDF 1.2: defensin	5′-TCATGGCTAAGTTTGCTTCC-3′ 5′-CACACGATTTAGCACCAAAGA-3′
*Nt* Hsr203J	5′-AGGAAGTATCCGGCTGGCTTAGA-3′ 5′-GAAGTAGTCATGGGGTGGGACTG-3′
*Nt* β-1,3 Glucanase (Glu)	5′-GCCAGATTTCTCTCCCCTATTCTC-3′ 5′-ACTCTCGGACACAACAATCCCTAC-3′
*Nt* Phenylalanine ammonia-lyase (PAL)	5′-GGACAAGGGCAGCTATGCTAGTTA-3′ 5′-CATTGAGGGTCTCACCATTAGGTC-3′
*At* β-actin	5′-TGCTCTTCCTCATGCTAT-3′ 5′-ATCCTCCGATCCAGACACTG-3′
*Nt* 26S rRNA	5′-CACGGACCAAGGAGTCTGACAT-3′ 5′-TCCCACCAATCAGCTTCCTTAC-3′
*Botrytis cinerea* β-actin	5′-TCCAAGCGTGGTATTCTTACC C-3′ 5′-TGGTGCTACACGAAGTTCGTTG-3′
16S rRNA: 27F and 1492R	5'-AGAGTTTGATCCTGGCTCAG-3' 5'-GGTTACCTTGTTACGACTT-3'

### Quantification of plant defense gene expression

Leaves from *N. tabacum* and *A. thaliana* plants were collected 48 h after the spray application of HKEB. Additionally, leaves from *A. thaliana* plants were collected 24, 48 and 72 h after the spray application of HKEB. Plants treated with water and mock-treated plants were used as controls. Total RNA was isolated using an RNeasy kit (Qiagen), and cDNA was synthesized using oligo-dT primers and a SuperScript III kit (Invitrogen, Carlsbad, CA, USA). Real-time quantitative PCR was performed using a Rotor-Gene Q PCR machine (Hilden, Germany) and the QuantiTect SYBR Green PCR Kit (Qiagen). The primer sequences are provided in Table 1. The *N. tabacum* 26S rRNA and *A. thaliana* β-actin genes were used as internal controls during RT-qPCR gene quantification. The reaction conditions for the real-time PCR were as follows: an initial denaturation step at 95 °C for 15 min, followed by denaturation at 95 °C for 15 s; an alignment step for 30 s at 60 °C; and an extension step for 30 s at 72 °C for 40 cycles. Relative gene expression was determined as mean normalized expression using *Q-Gene* software^88^.

### Identification of new genes using RNA sequencing

Arabidopsis plants were treated with HKEBs. Leaves were collected from five plants 48 h after the spray application. Mock-treated plants were used as controls. Total RNA was extracted using RNeasy Midi Kit (Qiagen), and the concentration of total RNA was determined using spectrometry. Three replicates of the treated and control samples were used per group. After extracting the total RNA, eukaryotic mRNA was enriched using oligo (dT) beads. The samples were sequenced using an Illumina HiSeq™ 2000 instrument by Gene Denovo Biotechnology Co.

High-quality reads were processed using a Perl script, and the differentially expressed genes were identified using the edgeR package (www.r-project.org/). Genes with a fold change in expression ≥ 2 were considered significant differentially expressed genes. Gene ontology (GO)^89^ and Kyoto Encyclopedia of Genes and Genomes (KEGG)^90^ pathway enrichment analyses were used to characterize the differentially expressed genes. GO functional annotations were obtained from the nonredundant annotation results. In addition, the GO annotations were analyzed using Blast2GO software^91^.

### Evaluation of defense responses against pathogens

To compare the effects of HKEBs on the control of pathogens in different plants, experiments were conducted on *N. tabacum* and *A. thaliana* plants that were previously inoculated with *B. cinerea* and *Pst*, respectively^81,82^. The effect of the HKEBs was evaluated as follows:

- Effect of HKEBs in *N. tabacum* inoculated with *B. cinerea*: Tobacco plants were sprayed with the *B. cinerea* strain in 200 ml of solution (sterile water + pathogen) at a density of 500,000 conidia/ml. Inoculated plants were grown under a transparent cover to maintain high humidity for 24 h. After inoculation with this pathogen, the plants were treated with HKEBs 24 and 72 h post-inoculation with the pathogen. Disease symptoms were evaluated 1 week post-inoculation. Additionally, *in planta* fungal growth was evaluated using the relative expression of the *B. cinerea* β-actin gene (Table 1) 1 week post-inoculation. Plants treated with water and mock-treated plants were used as controls. Total RNA was isolated using an RNeasy kit (Qiagen), and cDNA was synthesized using oligo-dT primers and a SuperScript III kit (Invitrogen). Real-time quantitative PCR was performed using a Rotor-Gene Q PCR machine (Qiagen) and the QuantiTect SYBR Green PCR Kit (Qiagen). The reaction conditions for the real-time PCR were as follows: an initial denaturation step at 95 °C for 15 min, followed by denaturation at 95 °C for 15 s; an alignment step for 30 s at 60 °C; and an extension step for 30 s at 72 °C for 40 cycles. Relative gene expression was determined as mean normalized expression using Q-Gene software^88^.
- Effect of HKEBs in *A. thaliana* inoculated with *B. cinerea*: Arabidopsis leaves were inoculated (10 μl of spore suspension placed on top of the leaf) at a density of 500,000 conidia/ml^81^. Inoculated plants were grown under a transparent cover to maintain high humidity. The final evaluation was performed 3 days later. The lesion diameter and symptoms were measured 3 days post-inoculation^81^. Plants treated with water and mock-treated plants were used as controls.
- Effect of HKEBs in *A. thaliana* inoculated with *Pst*: the *P. syringae* pv. tomato DC3000 strain was sprayed onto Arabidopsis leaves at a concentration of 5 × 10^8^ cfu/ml in water with 0.02% Silwet L-77^82^. Bacterial counting was performed on seven leaves with three replicates by surface sterilization with 70% ethanol at 1, 2, 3 and 4 days post-inoculation^82^. Plants treated with water and mock-treated plants were used as controls.

### Functional evaluation of HKEBs in Arabidopsis mutant plants

This experiment was conducted to determine the defense reactions of HKEB in different Arabidopsis mutants.

The Arabidopsis mutant plants were previously inoculated with *B. cinerea* and *Pst* pathogens^81,82^. Each mutant plant was sprayed with HKEB, and mock-treated plants were used as controls. The concentration of HKEB and pathogen inoculation procedures were as previously described protocol.

### Biochemical characterization of HKEBs using high-performance liquid chromatography mass spectrometry (HPLC/MS)

The sample powder was dissolved in 1 ml of a methanol–water (1:1) solution. Then, the sample was extracted with ethyl acetate three times and vacuum dried. After the ethyl acetate phase was dried, the residue was dissolved in a solution of methanol and water (1:1). Both samples were injected (20 µl) into an HPLC RP-C18 column for HPLC analysis. The HPLC conditions were as follows: methanol:water = 7:3, detection wavelength, 254 nm; detectors, time of flight; and ion sources, electrospray ionization (EI). The identification of the different compounds was performed using MassBank Norman according to the *m/z* values.

### Statistical analyses

All the assays were performed three times with five or ten replicates of each group, and the values presented in graphs/tables are means ± standard errors of the means. Statistically significant differences among the mean values were determined using a t test and/or ANOVA at P < 0.05. P values < 0.05 were considered statistically significant. Data were analyzed and processed using GraphPad Prism software (La Jolla, CA, USA).

## Supplementary Information

Supplementary Information 1.

Supplementary Information 2.

## Data Availability

The authors declare that all data supporting the findings of this study are available within the article and its Supplementary Information files or are available from the corresponding author upon request.
